# Review on Cardiorespiratory Complications after SARS-CoV-2 Infection in Young Adult Healthy Athletes

**DOI:** 10.3390/ijerph19095680

**Published:** 2022-05-06

**Authors:** Sofia Romagnoli, Agnese Sbrollini, Ilaria Marcantoni, Micaela Morettini, Laura Burattini

**Affiliations:** Department of Information Engineering, Università Politecnica delle Marche, 60131 Ancona, Italy; s.romagnoli@pm.univpm.it (S.R.); a.sbrollini@staff.univpm.it (A.S.); i.marcantoni@staff.univpm.it (I.M.); m.morettini@univpm.it (M.M.)

**Keywords:** SARS-CoV-2, COVID-19, sport, cardiovascular system, respiratory system

## Abstract

This review analyzes scientific data published in the first two years of the COVID-19 pandemic with the aim to report the cardiorespiratory complications observed after SARS-CoV-2 infection in young adult healthy athletes. Fifteen studies were selected using PRISMA guidelines. A total of 4725 athletes (3438 males and 1287 females) practicing 19 sports categories were included in the study. Information about symptoms was released by 4379 (93%) athletes; of them, 1433 (33%) declared to be asymptomatic, whereas the remaining 2946 (67%) reported the occurrence of symptoms with mild (1315; 45%), moderate (821; 28%), severe (1; 0%) and unknown (809; 27%) severity. The most common symptoms were anosmia (33%), ageusia (32%) and headache (30%). Cardiac magnetic resonance identified the largest number of cardiorespiratory abnormalities (15.7%). Among the confirmed inflammations, myocarditis was the most common (0.5%). In conclusion, the low degree of symptom severity and the low rate of cardiac abnormalities suggest that the risk of significant cardiorespiratory involvement after SARS-CoV-2 infection in young adult athletes is likely low; however, the long-term physiologic effects of SARS-CoV-2 infection are not established yet. Extensive cardiorespiratory screening seems excessive in most cases, and classical pre-participation cardiovascular screening may be sufficient.

## 1. Introduction

In December 2019, the novel “Severe Acute Respiratory Syndrome CoronaVirus 2” (SARS-CoV-2) was identified in Wuhan, China. SARS-CoV-2 is responsible for the Coronavirus disease 2019 (COVID-19) [1,2]. On 11 March 2020, the World Health Organization (WHO) declared the ongoing COVID-19 pandemic [3]. The primary clinical manifestation of COVID-19 is respiratory, causing acute respiratory disease. However, COVID-19 has multi-organ manifestations, frequently affecting the cardiovascular, renal, hematologic, musculoskeletal, gastrointestinal and nervous systems [2]. Indeed, COVID-19 is associated with a broad range of symptoms, including symptom clusters localized to the upper respiratory tract, general thoracic symptoms, gastrointestinal symptoms, and systemic symptoms. COVID-19 can manifest with different levels of severity, i.e., it can be asymptomatic, mild, moderate, or severe (requiring hospitalization) [2]. Especially, the elderly and patients with comorbidities can develop severe symptoms with poor outcomes, including death. Young and healthy subjects are less likely to develop moderate to critical symptoms due to COVID-19, remaining mainly asymptomatic or mildly symptomatic. Currently, the long-term complications of SARS-CoV-2 infection remain unclear [4,5]. Evidence of a severe spectrum of cardiovascular complications was apparent in a small sample size of hospitalized patients. This evidence could be affected by the potential long-term adverse effects of some therapeutic modalities that were implemented [6]. Therefore, the prevalence of cardiorespiratory complications after SARS-CoV-2 infection is not clearly defined in young adults and healthy populations (i.e., in groups of people in a state of complete physical, mental and social well-being [7]) with asymptomatic or mildly symptomatic manifestations [5,8].

With the term athletes, we indicate young and healthy subjects who place a high demand on their cardiorespiratory system due to a high level of physical activity. During the acute phase of the COVID-19 pandemic, several types of physical activity were suspended or postponed to both protect athletes’ health and limit the viral spread. However, when the infection diffusion decreased enough to allow resuming such activities, questions about the correct strategy to safely manage the return to play (RTP) of athletes were raised [5,6]. Indeed, there was a significant concern that subclinical cardiorespiratory complications due to COVID-19 could influence athletes’ physical performance and constitute a trigger for exercise-related clinical complications, including sport-related sudden cardiac death [5,6]. These concerns have doubted the diagnostic performance of actual pre-participation screening modalities, leading to the creation of RTP recommendations for athletes who have recovered from COVID-19 [4,5,6,8,9,10,11]. Several expert position statements and RTP recommendations have been published to guide athletes after SARS-CoV-2 infection [4,5,6,8,9,10,11], but they are mainly based on expert opinions and not on scientific findings [2,3,12]. The debate on the need of advanced cardiorespiratory screening in athletes after SARS-CoV-2 infection is still open because of the cost and difficulties (practical and emotional) in accessing the tests, especially in regions where COVID-19 prevalence is high and the number of athletes requiring it is large [3,8].

This review analyzes the scientific data published in the first two years of the pandemic relative to complications after SARS-CoV-2 infection in young adult healthy athletes. Here, the condition of health mainly refers to physical aspects, indicating absence of disease or infirmity. The aim was to report the symptomatology and the clinical complications observed after SARS-CoV-2 infection in young adult healthy athletes.

## 2. Materials and Methods

The literature search and method reporting performed here followed the PRISMA guidelines for systematic reviews and studies that evaluate healthcare interventions [13]. Discrepancies in studies selection and quality assessment were resolved after joint article review and discussion.

### 2.1. Literature Search Strategy

A systematic literature search was conducted in three electronic bibliographic databases, namely, PubMed, Scopus, and Web of Science, and in the organizational COVID-19 database of the WHO containing global research data on COVID-19. The literature was screened in the period from January 2020 to December 2021.

The roots ‘Athlet’ and ‘Sport’ were used to search for studies in the sport field. The roots ‘cardi’ and ‘respirat’ were used to search for studies on the cardiovascular system and respiratory system, respectively. The virus name ‘SARS-CoV-2’ and the related disease ‘COVID-19’ were used to search for studies related to the current pandemic. The search terms were organized into three concepts:

1. Athlet *, Sport *;
* cardi *, respirat *;
3. SARS-CoV-2, COVID-19.

Terms within each concept were combined with the Boolean operator ‘OR’ and then combined with the Boolean operator ‘AND’. ‘Title’ was used as a limit for the field of search, while English language was used as a limit to filter the documents. Only in the organizational database, the search query was constructed with the first two concepts, since the WHO database already contains only documents addressed by terms of the third concept (‘SARS-CoV-2’ and ‘COVID-19’).

### 2.2. Selection of Studies

Documents (research papers and letters) were imported into the Mendeley reference management system for duplicate removal. Titles were analyzed to include only documents about clinical complications after SARS-CoV-2 infection and COVID-19 syndrome in young adult athletes. Documents were excluded if related to sport industry management, sociological and psychological aspects of isolation and remote sports education. Research papers for which the abstract was not available were also excluded.

The eligibility criteria for abstract analysis were:

- Mean age of the athletes’ population greater than 18 years but lower than 35 years;
- Population of athletes with previous SARS-CoV-2 infection;
- Study on clinical complications after SARS-CoV-2 infection.

A full-text review was performed for both research articles and research letters describing observational or descriptive studies on clinical complications after SARS-CoV-2 infection and COVID-19 syndrome. Full text articles were excluded if evaluated of low quality according to the Joanna Briggs Institute Critical Appraisal tools checklist [14].

### 2.3. Data Analysis

Data were collected and analyzed in Microsoft Excel. Each study was described in terms of study design, athletes’ population characteristics (number, type, age, sex, ethnicity, anthropometric data and symptoms), clinical tests for screening and time between infection and clinical screening. Additionally, the populations were described in terms of practiced sports and main predictors of infection outcome in relation to age, sex, ethnicity, anthropometric data and symptoms. Continuous features (age, anthropometric data, time between infection and screening) were reported as mean ± standard deviation (SD) if normally distributed or as median and interquartile range (IQR) otherwise. Categorical features (sports, symptoms, sex and ethnicity) were reported as counts, ratio and/or percentage. Eventually, the subjects were classified as asymptomatic and symptomatic; the symptoms were characterized based on their severity, type and body region involved.

### 2.4. Quality Appraisal and Study Limitation Assessment

Quality appraisal was performed by two authors, independently, by assessing data quality and risk of bias using the Joanna Briggs Institute Critical Appraisal tools checklist [14].

Limitation of the selected studies was assessed based on the presence of control populations, which could consist of athletes with no SARS-CoV-2 infection, non-athletes with no SARS-CoV-2 infection, athletes with screening before SARS-CoV-2 infection (Pre-SARS-CoV-2 infection screening) and athletes with screening after infection by different respiratory viruses.

## 3. Results

**Figure 1:**
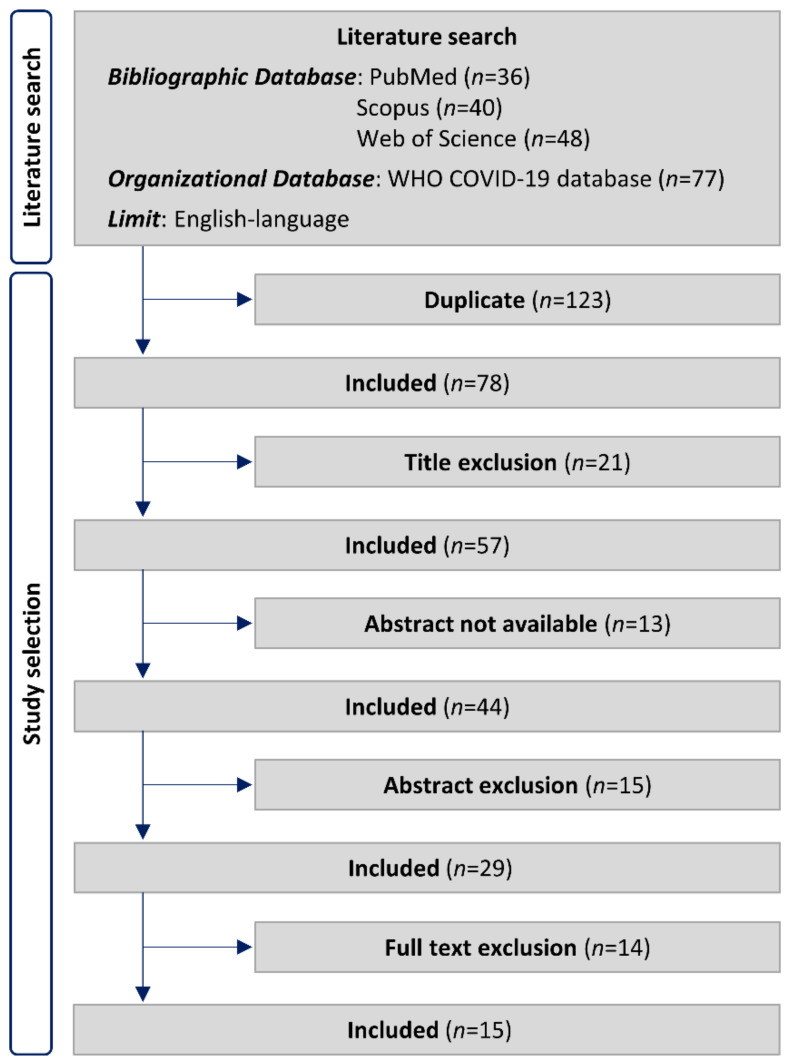
Performed literature search and study selection.

Overall, 201 studies were identified in the bibliographic and organizational databases; of these, 123 were duplicate, so that 78 were left for further analysis. After title, abstract and text analysis (the answers to Joanna Briggs Institute Critical Appraisal tools checklist [14] for quality appraisal are reported in the Supplementary Materials), 15 studies were selected [15,16,17,18,19,20,21,22,23,24,25,26,27,28,29]. Figure 1 depicts the entire processes of literature search and study selection.

**Figure 2:**
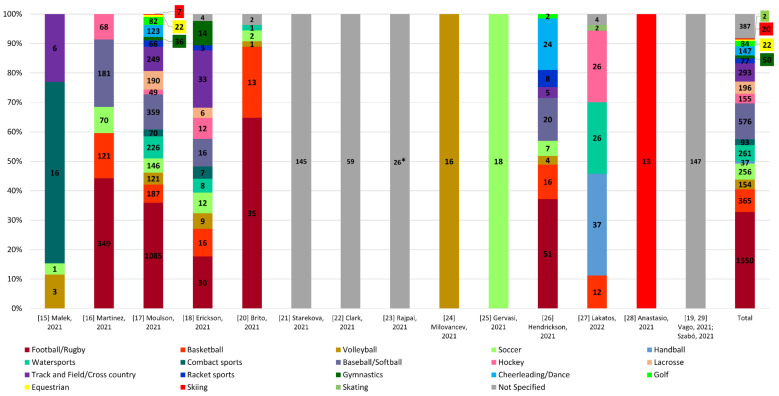
Column diagram of sport categories with counts and cumulative frequencies for each study and for the cumulative population of athletes. Some sport categories include two or more sport disciplines with similarities or common origins. Specifically, the football/rugby category includes football (1537) and rugby (13); the baseball/softball category includes baseball (479) and softball (97); the track-and-field/cross-country category includes track and field (n.a.) and cross country (n.a.); the racket sports category includes tennis (67) and squash (10); the watersports category includes swimming/diving (158), crew (54), water polo (37), sailing (10) and synchronized swimming (2); the combat sports category includes wrestling (77), fencing (15) and judo (1); finally, the cheerleading/dance category includes cheerleading (n.a.) and dance (n.a.). Study [29] includes the 12 athletes of study [19]. ‘*’ means that counts were not provided in the study and are consequently considered as not specified in the review.

Table 1 reports a description of the 15 selected studies. The athlete population of study [29] included the 12 athletes of study [19]; here, both studies [19,29] were reported but the provided common information was not duplicated in our analysis. Overall, the selected studies involved 4725 athletes, of which 3438 (73%) were males, and 1287 (27%) were females. Male athletes were prevalent in all studies (female athletes were absent in [24,25]) with the exception of [15,19,22]. Athletes were practicing 19 sport categories, some of which including two or more sport disciplines with similarities or common origins (Figure 2).

The most populated category was the football/rugby category, counting 1550 athletes; the least populated category was the skating category, counting 2 athletes.

**Figure 3:**
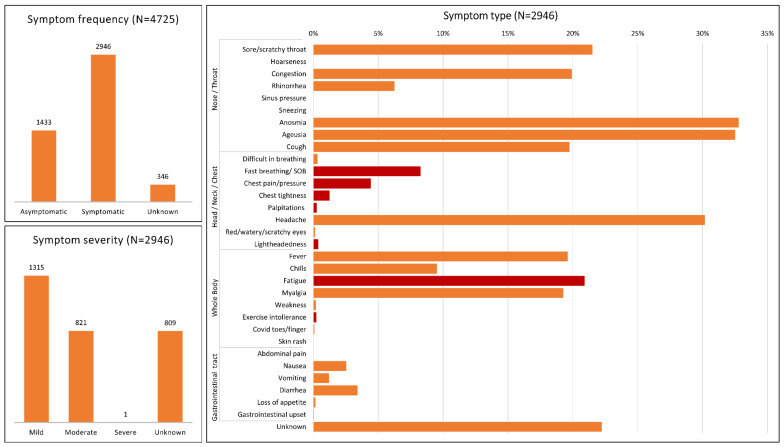
Report of symptoms. Up-left panel shows the distribution of asymptomatic and symptomatic athletes for the cumulative population of athletes (“unknown” indicates subjects who did not report the occurrence of symptoms). Bottom-left panel shows the distribution of symptom severity for the symptomatic athletes. Right panel shows the percentage of symptomatic athletes who reported a specific body-related symptom. Please note that an athlete could indicate the occurrence of one or more symptoms or could indicate or not the body region related to the symptom (“unknown” refers to symptoms for which information related to the body region was not provided). Orange and red bars refer to non-cardiopulmonary and cardiopulmonary symptoms, respectively.

In total, 4379 (93%) athletes released information about symptoms (Figure 3); 1433 (33%) of them declared to be asymptomatic, whereas the remaining 2946 (67%) reported the occurrence of symptoms with mild (1315; 45%), moderate (821; 28%), severe (1; 0%) and unknown (809; 27%) severity. The number of symptomatic patients was higher than the number of asymptomatic patients in almost all studies (except for [23,28]). A big variety of symptom types involving different body regions were reported, even though the most common were anosmia, ageusia and headache, which were reported by 33%, 32% and 30% of symptomatic athletes, respectively. Overall, cardiopulmonary symptoms, including fast breathing and SOB, chest pain, pressure and tightness, palpitations, lightheadedness, fatigue and exercise intolerance affected 1048 (36%) symptomatic athletes, with fatigue being the most common cardiorespiratory symptom (21%). Table 2 reports the occurrence of cardiorespiratory abnormalities detected with each screening test and confirmed cardiac inflammations, classified as myocarditis, pericarditis or other myopericardial inflammations. Cardiac magnetic resonance was the test that identified the largest number of cardiorespiratory abnormalities (15.7%). Among the confirmed inflammations, myocarditis was the most common (0.5%).

**Figure 4:**
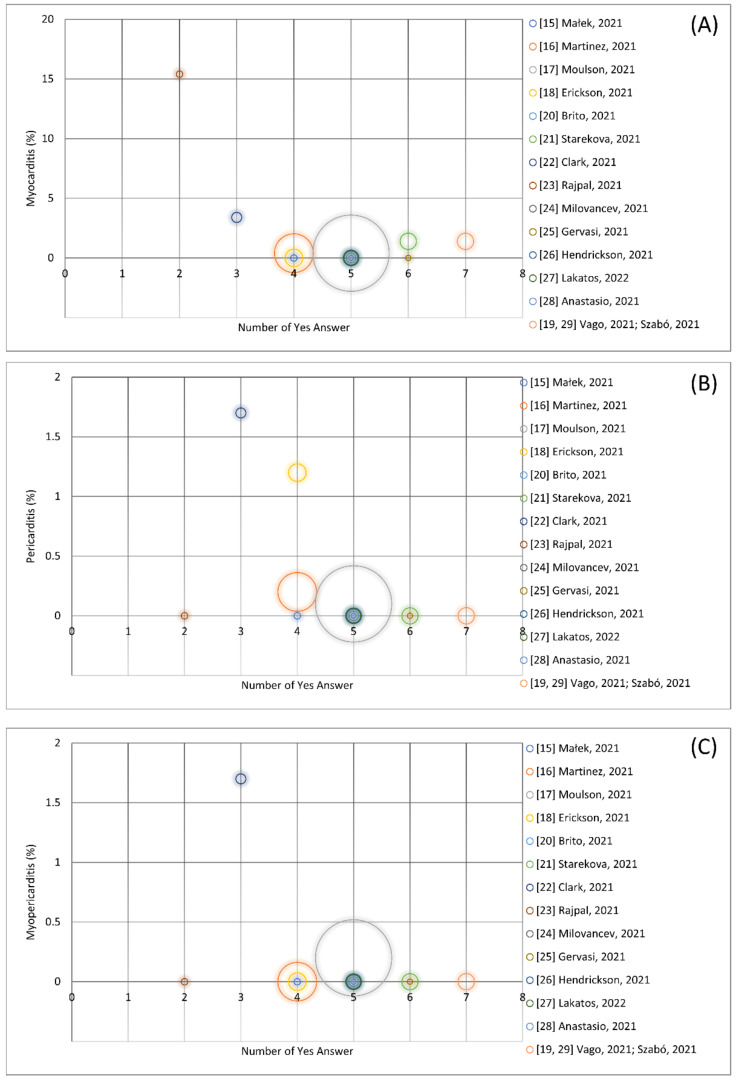
Total amount (percentage, %) of confirmed myocarditis (panel (**A**)), pericarditis (panel (**B**)) and myopericarditis (panel (**C**)) stratified by the number of Yes answers to the quality appraisal checklist and the size of the study populations represented by the areas of the circles.

When relating the occurrence of confirmed inflammation to the results of quality appraisal and size of the population (Figure 4), it emerged that the high percentage of cardiac inflammations derived from studies with low quality (low number of Yes answer to the quality appraisal checklist) and small population size.

Overall, 12 (0.2%) athletes needed hospitalization [15,16,17]; none of them required drug therapy, and no study reported previous comorbid conditions.

Table 3 reports the limitation of the studies, which was assessed based on the presence of control populations. Control populations composed of athletes or non-athletes not affected by SARS-CoV-2 were present in 7 (47%) and 3 (20%) studies out of 15, respectively. Only 2 (13%) studies could perform an intra-subject comparison of the screening results before and after the SARS-CoV-2 infection. Finally, no study could perform an intra-subject comparison of the screening results after the SARS-CoV-2 infection and infections due to different respiratory viruses.

## 4. Discussion

This review systematically analyzed the scientific data relative to complications after SARS-CoV-2 infection in athletes in order to report symptomatology and clinical complications after SARS-CoV-2 infection in young adult healthy athletes. Overall, 15 studies were included in the review. Selection was based on the language of the reports (English) and on athlete age (between 18 years and 35 years). Being English the language mostly used in science, we think that the choice of English as the only language for study selection had no impact on the study conclusions and, at the same time, makes the study easily understandable and repeatable. The age criterion was considered because subjects older than 35 years are likely less active and with a higher risk of comorbidities than young adult subjects [18]. Additionally, the causes of sport-related cardiac complications and death are known to change with athletes age, with a greater prevalence of cardiomyopathy and congenital coronary artery disease in young and middle-aged athletes, and of coronary atherosclerosis in older athletes [30,31,32].

Data from 4725 athletes, mainly males (78%), were analyzed and resulted very heterogeneous (Table 1, Figure 2). The athlete population included student, collegiate, competitive, tactical, professional, and elite athletes practicing 19 sport categories, for a total of 28 sports disciplines. All studies had an observational or descriptive design; still, they could be retrospective, prospective, cross-sectional, cohort, single- or multicenter, or case series. Most of them did not include a long-term clinical follow-up, which may be helpful to assess the prognosis of screening findings in athletes [15,20,21,23,25]. Only one study [29] presented a follow-up at about 232 days after infection. A positive test for SARS-CoV-2 virus was the common inclusion criterion among all studies. However, some studies involved athletes who resulted positive according to the polymerase chain reaction test or to the antibody test [15,16,17,18,19,20,21,22,23,24,25,26,27,28,29]. Some athletes were asymptomatic, other experienced various types of symptoms. Cardiorespiratory evaluation was always performed on athletes who already resulted negative but by performing different tests, namely, a laboratory test (including the analysis of troponin I, N-terminal pro-B-type natriuretic protein, high-sensitivity troponin T, high-sensitivity troponin I, and erythrocyte sedimentation rate), electrocardiography, echocardiography, cardiac magnetic resonance, a pulmonary function test and a cardio-pulmonary exercise test. The time between the positive test for SARS-CoV-2 and the cardiorespiratory screening was very variable among studies. This wide heterogeneity of athlete data allows a generalization of the obtained clinical information for RTP protocol optimization and obtaining insight into the effect of SARS-CoV-2 infection on the general young healthy population. However, the time course of COVID-19-associated cardiovascular complications is not understood. Thus, the optimal time to perform a screening remains unknown [21], and cardiorespiratory damages may remain undetected if the screening is performed too early [16].

Most athletes were symptomatic (67%), even though generally with a mild or moderate symptoms. Thus, a severe symptomatology appears rare in athletes (only 1 case out of 4725 athletes in our study). Symptom classification was difficult due to the existence of different guidelines to classify symptoms, leading to inhomogeneous reports among studies. Indeed, some studies classified symptoms based on their severity [15,17,18,20,21,22,23,24,25,26,27,29], while others based on the involved body region [17,18,19,20,21,24,25,26,27]. In general, mild to moderate nose and throat symptoms seemed to have the highest incidence in athletes (Figure 3), analogously to what observed in the adult general population mildly or moderately affected by SARS-CoV-2 infection [18]. 

Electrocardiography is a simple, affordable and thus common test for the non-invasive assessment of cardiac abnormalities. In sport, it is frequently included in the pre-participation screening protocols and may contribute to prevent sport-related sudden cardiac death [18]. In the studies analyzed here, electrocardiographic abnormalities were assessed following the international consensus criteria; the determined rate of electrocardiographic abnormalities (0.9%) was lower than that obtained using the international consensus criteria (1.8%) [33] and the Stanford criteria (6.6%) [34], respectively, in athletes never affected by SARS-CoV-2. This unexpected finding, however, may be due to the fact that some studies considered here did not report minor ECG abnormalities [21]. Consequently, the suitability of the electrocardiographic test for the detection of subclinical inflammatory heart diseases related to SARS-CoV-2 infection in young adult athletes remains unclear. In this context, also characterized by a substantial lack of reference athletes’ electrocardiograms [35], serial electrocardiography may be a potentially useful tool to detect a newly emerging cardiac pathology in athletes recovered from SARS-CoV-2 infection, especially if automatically performed using algorithms based on artificial intelligence [36]. Indeed, serial electrocardiography is an intra-subject evaluation consisting in the comparison of a newly made electrocardiogram with a previously made one to look for possible changes due to a worsening in the subject’s cardiac health status. Thus, by comparing the athlete’s electrocardiogram obtained during the pre-participation screening to that obtained after recovering from COVID-19, electrocardiographic changes (and thus cardiac complications) due to SARS-CoV-2 infection could be revealed.

In our study, cardiac imaging indicated a higher cardiac involvement due to SARS-CoV-2 (up to 15.7% for cardiac magnetic resonance; Table 2) than electrocardiography. The long-term clinical relevance of these findings remains unclear, given the absence of follow-up screening. The imaging test that detected more abnormalities was cardiac magnetic resonance. In most studies, it was not performed in asymptomatic athletes or in or mildly symptomatic athletes with normal first-line tests (troponin, electrocardiography, echocardiography) [15,16,17,18,19,21,25], so that the true prevalence of subclinical cases may result underestimated. Only two studies suggest performing (or performed) cardiac magnetic resonance in all athletes to diagnose cardiac inflammation, which may be missed with normal first-line tests [20,22]. In general, lack of normative athlete data, financial costs, and difficulties in accessing the test, suggest considering cardiac magnetic resonance as a clinically indicated and selective downstream test, rather than a first-line test for widespread screening [16].

Cardio-pulmonary exercise testing suggests a worsening of the respiratory function in SARS-CoV-2-positive athletes [15,18,25,28] but, in most cases, it is not clear if this finding is to be attributed to the detraining period rather than to COVID-19-related pulmonary damage. One study [28] suggested that the reduction in aerobic capacity measured by the test is related to a fitness reduction characterized by an early switch to anaerobic metabolism. Despite its simplicity and affordability, spirometry was rarely performed in athletes as a screening test (only in [25], as part of the cardio-pulmonary exercise testing).

In all studies analyzed here, symptoms, sex, ethnicity and anthropometric data were considered as predetermined predictors of clinical outcomes. Symptom burden should be carefully considered when screening SARS-CoV-2-positive athletes [16,37], since symptomatic athletes seem to present a greater myocardial involvement than asymptomatic athletes [18,20]. Females seems to have a more benign clinical course of the disease than males [17,18,23], even though this suggestion is not confirmed by all studies [15,19,22]. Given the heterogeneity of the data, no insight on clinical complications after SARS-CoV-2 infection related to ethnicity could be derived here, even though the literature suggests that individuals from minority ethnic backgrounds are at increased risk of adverse SARS-CoV-2 clinical outcomes [38]. Finally, the anthropometric data do not seem to be a predictor of major COVID-19 complications [18], possibly because athletes are in general more in shape that the general population (e.g., the body mass index is typically normal in athletes).

The lack of control populations, which are fundamental to assess the cardiorespiratory complications specifically due to SARS-CoV-2 infection, is the main limitation of most studies. The lack of SARS-CoV-2-negative control athletes in the studies may be due to pandemic restrictions, while less explainable is the lack of baseline pre-infection screening, especially for the tests included in the pre-participation screening. The few studies with control populations (Table 3) involved few tens of athletes each, and did not allow assessing cardiovascular complications in athletes due to COVID-19. Future studies with control populations are needed to better distinguish potential COVID-19-associated cardiac pathologies from exercise-related cardiac adaptation.

## 5. Conclusions

The low degree of symptom severity and the low rate of cardiac abnormalities observed here seems to suggest that the risk of significant cardiorespiratory involvement in young adult athletes is likely low; however, the long-term physiologic effects of SARS-CoV-2 infection are not established yet. According to the analyzed studies, extensive cardiorespiratory screening seems to be excessive in most cases, and classical pre-participation cardiovascular screening may be sufficient and may guarantee an optimize resource management by the health care system. Given the study limitations (including small sample size, heterogeneity of the data and existing reporting bias) this preliminary findings need to be confirmed by future studies. Thus, the results of this review should be definitely but critically considered in future position papers or consensus guidelines.

## Figures and Tables

**Table 1:** Descriptive summary of the analyzed studies. Continuous features (age, anthropometric data, time from infection) are reported in terms of mean ± standard deviation if normally distributed or as median and [interquartile range] or (range) if not normally distributed, as reported in the original studies. Categorical variables (sex and ethnicity) are reported as counts, ratio and/or percentage, as reported in the original studies.

Ref.	Study Design	Athletes Number and Type	Age (Years)	Sex M/F	Ethnicity	Anthropometric Data	Symptoms	Screening	Time from Infection (Days)
[15]	Retrospective Cohort	26 Elite	24[21–27]	5/21	Caucasian 26	*BSA* (kg/m^2^) 1.68(1.58–1.81)	Asymptomatic 6 Symptomatic 20 Unknown 0	Lab test ECG CMR	32[22–62]
[16]	Multicenter Retrospective Cross-sectional	789 Professional	25 ± 3	777/12	-	-	Asymptomatic 329 Symptomatic 460 Unknown 0	Lab test ECG ECHO CMR	19 ± 17 (3–156)
[17]	Prospective Multicenter Observational Cohort	3018 Competitive Collegiate	20 ± 1	2061/957	White non-Hispanic 1922 Black 829 White-Hispanic 87 Mixed 62 Other 80	*BMI* (kg/m^2^) Male: 27 ± 5 Female: 23 ± 3	Asymptomatic 887 Symptomatic 1789 Unknown 342	Lab test ECG ECHO CMR	Lab test 12[10–17] ECG 12[10–16] CMR 33[18–63] ECHO 15[11–25]
[18]	Retrospective	170 Collegiate	Male: 19.56 ± 1.51 Female: 19.44 ± 1.19	91/79	-	*Height* (m) Male: 1.84 ± 0.07 Female: 1.67 ± 0.07 *Weight* (kg) Male: 88.97 ± 18.01 Female: 63.96 ± 9.22 *BMI* (kg/m^2^) Male: 26.16 ± 4.47 Female: 22.69 ± 2.49	Asymptomatic 22 Symptomatic 147 Unknown 1	ECG	22.54 ± 14.20
[19]	-	12 Professional Elite	23[20–23]	2/10	-	-	Asymptomatic 2 Symptomatic 10 Unknown 0	Lab test CMR	Female: 17[17–19] Male: 67 and 90
[20]	Cross-sectional Observational	54 Student	19[19–21]	46/8	White 15 African-American 36 Others 3	*BMI* (kg/m^2^) Asymptomatic: 26.6[24.8–28.3] Symptomatic: 26.1[24.7–29.5]	Asymptomatic 16 Symptomatic 38 Unknown 0	Lab test ECG ECHO CMR	27[22–33]
[21]	Case series Retrospective	145 Competitive Student	19.6 ± 1.3	108/37	-	*Weight* (kg) 92 ± 25 *Height* (m) 1.84 ± 0.11 *BMI* (kg/m^2^) 27.0 ± 5.2 BSA (kg/m^2^) 2.1 ± 0.3	Asymptomatic 24 Symptomatic 118 Unknown 3	Lab test ECG ECHO CMR	Lab test 13[9–184] CMR 15[11–194]
[22]	-	59 Collegiate Tactical	20[19–21]	22/37	Non-white 6 Non-Hispanic 36	Weight (kg) 69[59–91] Height (cm) 173[164–188] BSA (kg/m^2^) 1.8[1.6–2.2]	Asymptomatic 13 Symptomatic 46 Unknown 0	ECHO CMR	21.5[13–37]
[23]	-	26 Competitive Collegiate	19.5 ± 1.5	15/11	-	-	Asymptomatic 14 Symptomatic 12 Unknown 0	Lab test ECG ECHO CMR	(11–53)
[24]	Cross-sectional	16 Professional	24 ± 4.5	16/0	Serbian 16	Weight (kg) 90 ± 8.9 Height (cm) 193.4 ± 9.9 BMI (kg/m^2^) 24.3 ± 2.4	Asymptomatic 0 Symptomatic 16 Unknown 0	ECG CPET	22 ± 7 training cessation 20.1 ± 4.7 training before testing
[25]	Cohort	18 Professional	22[21–27]	18/0	-	-	Asymptomatic 6 Symptomatic 12 Unknown 0	Lab test ECG ECHO CMR PFT	15 from recovery
[26]	Retrospective	137 collegiate	20(18–27)	93/44	Black 66 White 65 Hispanic 6	-	Asymptomatic 25 Symptomatic 112 Unknown 0	Lab test ECG ECHO CMR	16(12–34)
[27]	Prospective	107 Elite	23 ± 6	82/25	-	Height (cm) 182.9 ± 10.0 Weight (kg) 80.2 ± 15.3 BSA (m2) 2.0 ± 0.2	Asymptomatic 59 Symptomatic 48 Unknown 0	ECHO	22(17–25)
[28]	Retrospective	13 Elite	21 ± 5	10/3	Italian 13	Weight (kg) 67	Asymptomatic 13 Symptomatic 0 Unknown 0	ECHO CPET PFT	(28–42)
[29]	Observational	147 Elite	23[20–28]	94/53	-	BSA (m2) 2.0 ± 0.2	Asymptomatic 19 Symptomatic 128 Unknown 0	Lab test ECG CMR	32

CMR: Cardiac Magnetic Resonance; CPET: CardioPulmonary Exercise Testing; ECHO: ECHOcardiography; ECG: ElectroCardioGraphy; PFT: Pulmonary Function Test.

**Table 2:** Occurrence of cardiorespiratory abnormalities expressed as number of detected abnormalities over the total number of screening tests, and number of confirmed cardiac inflammation over the total population.

Ref.	Detected Abnormalities						Confirmed Inflammation		
	Lab Test	ECG	ECHO	CMR	PFT	CPET	Myocarditis	Pericarditis	Myopericardial
[15]	0/26 (0.0%)	0/26 (0.0%)	-	5/26 (19.2%)	-	-	0/26 (0.0%)	0/26 (0.0%)	0/26 (0.0%)
[16]	6/789 (0.8%)	10/789 (1.3%)	20/789 (2.5%)	9/27 (33.3%)	-	-	3/789 (0.4%)	2/789 (0.2%)	0/789 (0.0%)
[17]	24/2719 (0.9%)	21/2999 (0.7%)	24/2556 (0.9%)	21/317 (6.6%)	-	-	11/3018 (0.4%)	4/3018 (0.1%)	6/3018 (0.2%)
[18]	-	6/170 (3,5%)	-	-	-	-	0/170 (0.0%)	2/170 (1.2%)	0/170 (0.0%)
[20]	1/54 (1.8%)	1/54 (1.8%)	27/48 (56.2%)		-	-	0/54 (0.0%)	0/54 (0.0%)	0/54 (0.0%)
[21]	4/154 (2.6%)	0/154 (0.0%) *	0/154 (0.0%)	42/145 (29.0.%)	-	-	2/145 (1.4%)	0/145 (0.0%)	0/145 (0.0%)
[22]	-	-	-	23/59 (39.0%)	-	-	2/59 (3.4%)	1/59 (1.7%)	1/59 (1.7%)
[23]	0/26 (0.0%)	0/26 (0.0%)	0/26 (0.0%)	12/26 (46.1%)	-	-	4/26 (15.4%)	0/26 (0.0%)	0/26 (0.0%)
[24]	-	0/16 (0.0%)	-	-	-	0/16 (0.0%)	0/16 (0.0%)	0/16 (0.0%)	0/16 (0.0%)
[25]	1/18 (5.5%)	0/18 (0.0%)	0/18 (0.0%)	0/1 (0.0%)	2/18 (11.1%)	-	0/18 (0.0%)	0/18 (0.0%)	0/18 (0.0%)
[26]	4/137 (2.9%)	0/137 (0.0%)	2/137 (1.5%)	0/5 (0.0%)	-	-	0/137 (0.0%)	0/137 (0.0%)	0/137 (0.0%)
[27]	-	-	5/107 (4.7%)	0/5 (0.0%)	-	-	0/107 (0.0%)	0/107 (0.0%)	0/107 (0.0%)
[28]	-	-	0/13 (0.0%)	-	0/13 (0.0%)	0/13 (0.0%)	0/13 (0.0%)	0/13 (0.0%)	0/13 (0.0%)
[19,29]	6/133 (4.5%)	-	-	7/147 (4.7%)	-	-	2/147 (1.4%)	0/147 (0.0%)	0/147 (0.0%)
Overall	46/4056 (1.1%)	38/4389 (0.9%)	51/3800 ^§^ (1.3%)	119/758 ^§^ (15.7%)	2/31 (6.4%)	0/29 (0.0%)	24/4725 (0.5%)	9/4725 (0.2%)	7/4725 (0.1%)

* Absence of minor ECG abnormalities; ^§^ Ref. [20] not included; CMR: Cardiac Magnetic Resonance; CPET: CardioPulmonary Exercise Testing; ECHO: ECHOcardiography; ECG: ElectroCardioGraphy; PFT: Pulmonary Function Test.

**Table 3:** Limitation of the studies, assessed by checking the presence of control populations (“Yes” and “No” indicate the presence or absence of a control population, respectively).

Ref.	Athletes with No SARS-CoV-2 Infection	Non-Athletes with No SARS-CoV-2 Infection	Pre SARS-CoV-2 Infection Screening	Screening after Different Respiratory Viruses
[15]	No	No	No	No
[16]	No	No	No	No
[17]	No	No	No	No
[18]	No	No	No	No
[19]	Yes	Yes	No	No
[20]	Yes *	No **	No	No
[21]	No	No	No	No
[22]	Yes	Yes	No	No
[23]	No	No	No	No
[24]	No	No	No	No
[25]	Yes	No	Yes	No
[26]	No	No	No	No
[27]	Yes	No	No	No
[28]	Yes	No	No	No
[29]	Yes	Yes	Yes	No

* [20] did not include a control group for CMR testing ** [20] included a normal reference only for CMR.

## Data Availability

Not applicable.

## Supplementary Materials

The following supporting information can be downloaded at: https://www.mdpi.com/article/10.3390/ijerph19095680/s1, Extended query for literature search, Quality appraisal.
