# Supplementary material for: Review on Cardiorespiratory Complications after SARS-CoV-2 Infection in Young Adult Healthy Athletes
Source: Int J Environ Res Public Health. 2022 May 6;19(9):5680. doi: 10.3390/ijerph19095680 (PMC9101647; doi:10.3390/ijerph19095680)
Supplement: Supplementary file 1 [file ijerph-19-05680-s001.zip › Extended query for literature search.pdf]

Search query:

1) Pubmed

Search: (sport*[Title] OR athlet*[Title]) AND ("SARS-CoV-2"[Title] OR "COVID-19"[Title]) AND (*cardi*[Title] OR respirat*[Title]) Filters: English, from 2020 - 2021

2) Scopus

TITLE ((sport* OR athlet*) AND ({SARS-CoV-2} OR {COVID-19}) AND (*cardi* OR respirat*)) AND (LIMIT-TO (PUBYEAR , 2021) OR LIMIT-TO (PUBYEAR , 2020)) AND (LIMIT-TO (LANGUAGE , "English"))

3) Web of Science

TI=((sports* OR athlet*) AND (“SARS-CoV-2” OR “COVID-19”) AND (*cardi* OR respirat*)) and 2021 or 2020 (Publication Years) and English (Languages)

4) World Health Organization

ti:((sport* OR athlet*) AND (*cardi* OR respirat*)) AND la:("en") AND year_cluster:("2021" OR "2020")
