# Supplementary material for: Review on Cardiorespiratory Complications after SARS-CoV-2 Infection in Young Adult Healthy Athletes
Source: Int J Environ Res Public Health. 2022 May 6;19(9):5680. doi: 10.3390/ijerph19095680 (PMC9101647; doi:10.3390/ijerph19095680)
Supplement: Supplementary file 1 [file ijerph-19-05680-s001.zip › Quality_apprasial.pdf]

Table S1. Quality appraisal of cross-sectional studies according to the Joanna Briggs Institute Critical Appraisal tools checklists (Y=Yes, N=No, U=Unclear, NA=Not Applicable).

	**[16]**				**[20]**				**[24]**			
	**Y**	**N**	**U**	**NA**	**Y**	**N**	**U**	**NA**	**Y**	**N**	**U**	**NA**
**Were the criteria for inclusion in the sample clearly defined?**	X				X				X			
**Were the study subjects and the setting described in detail?**		X			X				X			
**Was the exposure measured in a valid and reliable way**	X				X				X			
**Were objective, standard criteria used for measurement of the condition?**	X					X			X			
**Were confounding factors identified?**		X				X				X		
**Were strategies to deal with confounding factors stated?**		X				X				X		
**Were the outcomes measured in a valid and reliable way?**	X				X				X			
**Was appropriate statistical analysis used?**		X			X					X		
**Total Answer**	4	4	0	0	5	3	0	0	5	3	0	0

Table S2. Quality appraisal of case series study according to the Joanna Briggs Institute Critical Appraisal tools checklists (Y=Yes, N=No, U=Unclear, NA=Not Applicable).

	**[21]**			
	**Y**	**N**	**U**	**NA**
**Were there clear criteria for inclusion in the case series?**		X		
**Was the condition measured in a standard, reliable way for all participants included in the case series?**	X			
**Were valid methods used for identification of the condition for all participants included in the case series?**	X			
**Did the case series have consecutive inclusion of participants?**	X			
**Did the case series have complete inclusion of participants?**	X			
**Was there clear reporting of the demographics of the participants in the study?**		X		
**Was there clear reporting of clinical information of the participants?**		X		
**Were the outcomes or follow up results of cases clearly reported?**	X			
**Was there clear reporting of the presenting site(s)/clinic(s) demographic information?**		X		
**Was statistical analysis appropriate?**	X			
**Total Answer**	6	4	0	0

Table S3. Quality appraisal of cohort studies according to the Joanna Briggs Institute Critical Appraisal tools checklists (Y=Yes, N=No, U=Unclear, NA=Not Applicable).

	[15]				[17]				[18]				[19]				[22]				[23]				[25]				[26]				[27]				[28]				[29]			
	Y	N	U	NA	Y	N	U	NA	Y	N	U	NA	Y	N	U	NA	Y	N	U	NA	Y	N	U	NA	Y	N	U	NA	Y	N	U	NA	Y	N	U	NA	Y	N	U	NA	Y	N	U	NA
**Were the two groups similar and recruited from the same population?**				X				X				X	X				X							X	X							X	X				X				X			
**Were the exposures measured similarly to assign people to both exposed and unexposed groups?**				X				X				X		X				X						X	X							X		X				X				X		
**Was the exposure measured in a valid and reliable way?**	X				X				X				X				X				X				X				X				X				X				X			
**Were confounding factors identified?**		X				X				X				X				X				X				X				X				X				X				X		
**Were strategies to deal with confounding factors stated?**		X				X				X				X				X				X				X				X				X				X				X		
**Were the groups/participants free of the outcome at the start of the study (or at the moment of exposure)?**	X						X				X		X						X				X		X						X		X				X				X			
**Were the outcomes measured in a valid and reliable way?**	X				X				X				X					X			X				X				X				X				X				X			
**Was the follow up time reported and sufficient to be long enough for outcomes to occur?**		X			X					X				X				X				X				X			X					X				X			X			
**Was follow up complete, and if not, were the reasons to loss to follow up described and explored?**		X			X				X					X				X				X				X			X					X				X			X			
**Were strategies to address incomplete follow up utilized?**		X				X				X				X				X				X				X				X				X				X				X		
**Was appropriate statistical analysis used?**	X				X				X				X				X					X			X				X				X				X				X			
**Total Answer**	4	5	0	2	5	3	1	2	4	4	1	2	5	6	0	0	3	7	1	0	2	6	1	2	6	5	0	0	5	3	1	2	5	6	0	0	5	6	0	0	7	4	0	0
